# Effect of guidewire on contribution of loss due to momentum change and viscous loss to the translesional pressure drop across coronary artery stenosis: An analytical approach

**DOI:** 10.1186/1475-925X-10-51

**Published:** 2011-06-10

**Authors:** Ehsan Rajabi-Jaghargh, Kranthi K Kolli, Lloyd H Back, Rupak K Banerjee

**Affiliations:** 1 School of Dynamic Systems, Mechanical Engineering Program, University of Cincinnati, 593 Rhodes Hall, PO Box 210072, Cincinnati OH, USA; 2 Jet Propulsion Laboratory, California Institute of Technology, Pasadena, CA, USA

**Keywords:** Throat length, throat diameter, guidewire, hyperemic flow, basal flow, viscous loss, mo-mentum change, diagnostic parameter, stenosis

## Abstract

**Background:**

Guidewire (GW) size and stenosis dimensions are the two major factors affecting the translesional pressure drop. Studying the combined effect of these parameters on the mean pressure drop (Δ*p*) across the stenosis is of high practical importance.

**Methods:**

In this study, time averaged mass and momentum conservation equations are solved analytically to obtain pressure drop-flow, Δ*p*-*Q*, curves for three different percentage area blockages corresponding to moderate (64%), intermediate (80%), and severe (90%) stenoses. Stenosis is considered to be axisymmetric consisting of three different sections namely converging, throat, and diverging regions. Analytical expressions for pressure drop are obtained for each of these regions separately. Using this approach, effects of lesion length and GW insertion on the mean translesional pressure drop and its component (loss due to momentum change and viscous loss) are analyzed.

**Results and Conclusion:**

It is observed that for a given percent area stenosis (AS), increase in the throat length only increases the viscous loss. However, increase in the severity of stenosis and GW insertion increase both loss due to momentum change and viscous loss. GW insertion has greater contribution to the rise in viscous loss (increase by 2.14 and 2.72 times for 64% and 90% AS, respectively) than loss due to momentum change (1.34% increase for 64% AS and 25% decrease for 90% AS). It also alters the hyperemic pressure drop in moderate (48% increase) to intermediate (30% increase) stenoses significantly. However, in severe stenoses GW insertion has a negligible effect (0.5% increase) on hyperemic translesional pressure drop. It is also observed that pressure drop in a severe stenosis is less sensitive to lesion length variation (4% and 14% increase in Δ*p *for without and with GW, respectively) as compared to intermediate (10% and 30% increase in Δ*p *for without and with GW, respectively) and moderate stenoses (22% and 48% increase in Δ*p *for without and with GW, respectively). Based on the contribution of pressure drop components to the total translesional pressure drop, it is found that viscous losses are dominant in moderate stenoses, while in severe stenoses losses due to momentum changes are significant. It is also shown that this simple analytical solution can provide valuable information regarding interpretation of coronary diagnostic parameters such as fractional flow reserve (FFR).

## 1. Background

Formation of stenosis in coronary arteries is the leading cause of myocardial infarction and death in United States [1], and therefore, accurate assessment of the stenosis severity is crucial to the interventional cardiologists. In interventional cardiology fractional flow reserve (FFR; the ratio of average pressure distal [*p_d _*] and proximal to stenosis [*p_a_*] measured at maximal flow (hyperemia)) and coronary flow reserve (CFR; the ratio of blood flow rates at hyperemic to basal condition) are measured to find the functional severity of coronary stenosis [2]. It can be noted that these diagnostic parameters (FFR and CFR) are either ratio of pressure drop or blood flow rate. However, recent studies [3,4] have proposed that the *combined use of translesional pressure drop and blood flow rate *can improve the functional assessment of stenosis severity. Accordingly, in the newly proposed diagnostic parameters translesional pressure drop is scaled either linearly or quadratically with flow rate. It may be noted that an appropriate choice of scaling factor can result in non-dimensional diagnostic parameters by including fluid properties (viscosity and density) and geometric information (diameter). Therefore, there is a need to analytically determine the appropriate scaling approach that can be applied to different ranges of stenoses severity and flow rates. An appropriate scaling approach can be determined and put into practice by exploring the pressure drop and its components (viscous losses [linear relation with flow rate] and losses due to momentum changes [quadratic relation with flow rate]) along the stenosis for different flow rates and stenoses severity.

Pressure drop across a stenosis is a function of blood flow rate and lesion anatomy (lesion dimensions and stenosis severity [AS]). In current clinical practice geometric (or anatomic) information can be obtained using bi-planar quantitative coronary angiography (QCA). Furthermore, the functional (hemodynamic) endpoints can be assessed using Doppler flow guidewire (GW) and/or piezoelectric pressure wires [4-6]. This study proposes a quick and inexpensive analytical approach that can potentially utilize the information from QCA and GW to evaluate the translesional pressure drop and thus, diagnostic parameters during the cardiac catheterization procedure.

In the catheterization lab FFR is the current clinical diagnostic gold standard for detecting the severity of stenosis. If FFR falls below 0.75, then it is clinically considered as an ischemic condition and the patient may be treated by coronary intervention (e.g. angioplasty). Brosh et al. [7] studied 63 patients suffering from coronary artery disease and found out that lesion length and in particular stenosis severity have significant impact on the FFR values of intermediate coronary stenoses. The effects of GW and vessel diameter along with percent area stenosis (AS) were also discussed in an *in vitro *study by De Bruyne et al. [8], where the lesion was modeled as an orifice. Numerical validation of pressure drop measured in *in vivo *experiments and GW flow obstruction effect was quantified by Banerjee et al. [9,10].

Pressure drop-flow, Δ*p-Q*, relation in the stenosis region has been studied by many researchers for a wide range of geometries [8,11-17] and flow rates [18-20]. However, there is not much analytical work, that can be used in clinical practice, to assess the combined effect of throat geometry and GW on transstenotic pressure drop. Thus, the goal of this study is to find the effect of the throat length, AS, and influence of GW on pressure drop and its components (viscous losses and losses due to momentum changes) across the stenosis. Studying the components of pressure drop would allow us to determine their contribution to the total pressure drop. Thus, in turn, will allow better scaling of diagnostic parameters and possibly improved quantification of coronary artery impairment in the cardiac catheterization lab.

## 2. Method

In this study, mean pressure drop is obtained analytically for moderate (64%), intermediate (80%), and severe (90%) stenoses using the approach proposed by Back et al. [21]. Effects of lesion length, GW insertion, and plaque severity on translesional pressure drop are analyzed. Details on stenosis configuration and mathematical formulation are presented below.

### 2.1 Geometry

**Figure 1:**
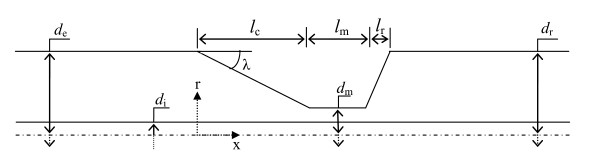
**Axisymmetric geometry of stenosis: showing converging, throat, and diverging regions**.

Pressure drop across the stenosis can be correlated with stenosis geometry (plaque severity and profile) [13,15], blood viscosity, and flow condition (basal or hyperemic). In this work, as shown in Figure 1, stenosis geometry is considered to be axisymmetric with trapezoidal profile. Lesion dimensions are obtained from pre- and post- coronary angioplasty data of 32 patients as reported by Wilson et al. [20]. Stenosis geometry is consisted of three distinct regions namely converging, throat, and diverging sections. Table 1 presents the dimensional characteristic for moderate (64%), intermediate (80%), and severe (90%) stenoses. All these area stenoses are associated to a clinically relevant case. The 64% and 90% area stenoses correspond to after and before coronary artery angioplasty, respectively [9,10,21,22]. The intermediate stenosis represents a clinically challengeable case from diagnostic viewpoint. FFR (ratio of average pressure distal (*p_d_*) and proximal (*p_a_*) to stenosis [FFR = *p_d _/p_a_*] under hyperemic condition) values for intermediate stenoses might be affected by the variation in pressure drop due to presence of GW which will be discussed in detail in the results section.

**Table 1:** Geometry of stenosis (All dimensions are in mm)

Geometry	*d_e _= d_r_*	*l_c_*	*d_m_*	*l_m_*	*l_m_/d_m_*	*l_r_*
	3.0	6.0	1.80	1.50	0.83	1.5
	
64%	3.0	6.0	1.80	3.00	1.67	1.5
	
	3.0	6.0	1.80	4.50	2.50	1.5

	3.0	6.0	1.35	0.75	0.57	1.5
	
80%	3.0	6.0	1.35	1.50	1.14	1.5
	
	3.0	6.0	1.35	2.25	1.70	1.5

	3.0	6.0	0.95	0.25	0.26	1.5
	
90%	3.0	6.0	0.95	0.50	0.52	1.5
	
	3.0	6.0	0.95	0.75	0.79	1.5

A coronary artery diameter of 3 mm and a constant converging length (*l_c_*) of 6 mm is assumed for all AS [20,21]. Previous studies have shown that pressure drop along the stenosis can be somewhat affected by the stenosis exit angle [23,24]. However, previous studies by Lipscomb et al. [13] have shown that pressure drop across the stenosis is not affected by varying the stenosis exit angle from 10° to 90°. This analytical approach doesn't consider the effect of exit angle which may have some effect on *Δp *and thus needs to be assessed in future studies. Constant diverging length (*l_r_*) of 1.5 mm is assumed for all AS. For a particular AS with throat diameter of *d_m_*, throat lengths (*l_m_*) are chosen such that the distal pressure remains within the physiological range (> 55 mmHg) [25]. Therefore, to satisfy this criterion, throat lengths for 90%, 80%, and 64% area stenoses are chosen in the ranges of 0.25 to 0.75 mm, 0.75 to 2.25 mm, and 1.5 to 4.5 mm, respectively. Moreover, proximal and distal diameters are assumed to be identical (*d_e _*= *d_r_*). Assuming a constant *l_c _*and *l_r_*, the total lesion length (*L *= *l_c _*+ *l_m _*+ *l_r_*) varies only with *l_m_*. Thus, it is possible to assess the effect of throat length on translesional pressure drop, with and without GW. Previous studies have used GW and catheters with large diameters (0.66 mm [18,26] to 1.4 mm [21]), however, in this study a GW diameter (*d_i_*) of 0.35 mm is considered, which is the most commonly used GW under current clinical practice. Pressure drop is calculated at each section (converging, throat, and diverging) separately. The analytical formulation is summarized in the following section.

### 2.2 Mathematical Model

The conservation of mass and momentum equations in their integral form are applied to find the relation between pressure drop, flow, and lesion geometry.

Mass balance equation:(1)

Momentum balance equation:(2)

After applying these equations to the geometry shown in Figure 1, mass and momentum balance equations can be written as follows:(3)(4)

The mean momentum balance equation can be obtained by integrating equation (4) over a period T of cardiac cycle:(5)

Where ū is the average axial velocity during the cycle and  is the energy correction factor based on the linear flow theory. The symbol (~) represents the time average of the corresponding parameters which will be dropped from the rest of equations in the paper. Therefore, as described above mean pressure drop along the stenosis is obtained under steady, laminar flow, and Newtonian fluid assumptions.

### 2.2.1 Pressure Drop Calculations

Stenosis geometry in general is comprised of converging, throat, and diverging sections. In the converging section mean blood flow velocity increases and flow accelerates. Therefore, in this region pressure drops due to both momentum change and viscous loss. As the flow advances towards the throat area, flow momentum varies due to entrance effects, however, in the current analytical approach the induced loss due to momentum change is considered to be negligible when compared to viscous loss. Further as the flow enters the diverging section due to the adverse pressure gradients, flow separates from the wall forming a recirculation zone near to the wall along with a high momentum stenotic jet in the center. The pressure recovery in this section is of the order of the throat's dynamic pressure and is estimated using a pressure recovery coefficient. Inserting the GW shifts the flow maximum velocity pocket towards the GW surface inducing high shear forces. Due to GW blockage effect loss due to momentum change also increases. To sum up, losses in different regions of a stenosis are dominated with either viscous or momentum change or both of them. Therefore, it is of interest to determine the contribution of loss due to momentum change and viscous loss to translesional pressure drop for different area stenoses.

*Pressure Drop across the Converging Region*. Integrating equation (5) along the converging length results in the mean pressure drop correlation across this region:(6)

where  is shear force integral with H defined as 

and , while  is defined as 

In these equations *r_o _*varies with axial length (*x*) as *r_o _*= *r_e _*- *x*tan(*λ*), where *λ*= tan^-1^((*r_e_*-*r_m_*)/*l_c_*) is the slope of converging section and *x *is calculated from the stenosis leading edge as shown in Figure 1. Flow rate is calculated using the following equation: *Q *= *ū_e_A_E _*=*ū_m_A_M_*, where *ū_e _*and *ū_m _*are, respectively, the average velocities at proximal and at the throat section of stenosis; while *A_E _*= (*A_e _*- *A_i_*) and *A_M _*= (*A_m _*- *A_i_*) represent the arterial cross-sections at corresponding regions, respectively.

*Pressure Drop across Throat Region*. GW can be inserted in the vessel either concentrically or eccentrically, however, it has been shown that the former results in maximum pressure drop along the throat [27]. The pressure drop for the concentric annular flow between the vessel and GW can be written as below:(7)

where  is the increase in flow resistance due to the presence of catheter, and depends only on the ratio of catheter to vessel radius (or diameter). In the absence of a catheter *r_i _*→ 0 and *F *→ 1, thus, reducing equation (7) to Poiseuille flow relation.

*Pressure Recovery across the Diverging Region*. In the divergent region mean flow decelerates resulting in flow separation which makes the flow field too complicated to be solved analytically. Distal to plaque, pressure recovery (Δ*p_rm_*) can be scaled by the throat dynamic pressure (), and can be written in the following form:(8)

where *C_pr _*is pressure recovery coefficient considering pressure changes due to both viscous losses and momentum changes. Ignoring wall friction, *C_pr _*can be obtained by only considering the pressure recovery due to momentum changes shown as . The basis of  relies on the assumption of negligible shear forces referred to as high Reynolds number limit [27], which in general leads to higher pressure recovery values as compared to *C_pr_*. Effect of viscous losses can be also included in  by multiplying it with a correction factor (*β*); . However, previous studies [22] have shown that for throat Reynolds numbers greater than 673 (which is the case for all hyperemic flow rates in this work) viscous losses are of minor importance and *β *tends to one; therefore pressure recovery can be properly assessed by only considering the momentum changes in flow [27]. The total mean pressure drop across the stenosis is then obtained by adding up equations (6), (7), and (8):(9)

Contribution of loss due to momentum change and viscous loss to the total pressure drop are shown in the following equations, respectively:(10)(11)

Equations (9) to (11) can be solved to obtain each component of pressure drop. Integral *I_s _*is solved numerically along the entire converging length using trapezoidal integration method. Pressure drops are calculated for different AS considering three different throat lengths for each stenosis in the absence and presence of GW. The Δ*p*-*Q *characteristic curves are obtained for each case and the contribution of loss due to momentum change and viscous loss to the total pressure drop are evaluated.

## 3. Results

Flow is assumed to be steady and laminar. Blood is treated as Newtonian fluid with the viscosity of 3.5 cP and density of 1050 kg/m^3^. Pressure drop variation with flow rate is obtained for different throat lengths and stenosis severity, considering the effect of GW. Throat lengths for severe (90%), intermediate (80%), and moderate (64%) stenoses vary from 0.25 to 0.75 mm, 0.75 to 2.25 mm, and 1.5 to 4.5 mm, respectively. Flow rate varies from basal to hyperemic in all the figures. Basal flow for all the cases is 50 ml/min, while the hyperemic flow rate is different for each of the cases considered in this study. The physiological cut off value of hyperemic flow rate has an inverse correlation with the flow resistance. That is, severe stenosis with the highest flow resistance, among other plaques, has the lowest hyperemic flow rate. The cut off values for hyperemic flow rates before insertion of GW for 64%, 80%, and 90% area stenoses are 180, 165, and 115 ml/min, respectively. However, in the presence of the GW these values reduce to 173, 150, and 85 ml/min, respectively. The cut off values for hyperemic flow rates in the absence of GW are chosen based on the pre- and post- angioplasty data of Wilson et al. [20], while the hyperemic flow rates in the presence of GW are adopted from the study of Roy et al. [28]. It should be noted that, all percentages presented for the comparison of pressure drops between with and without GW cases are obtained from pressure drops at their corresponding hyperemic flow rates.

### 3.1 Variation of Pressure Drop (Δ*p*) with Flow Rate (*Q*)

**Figure 2:**
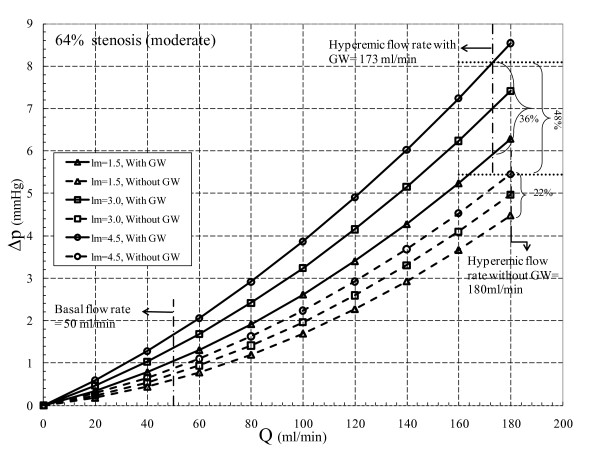
**64% area blockage Δp vs. Q characteristic**.

**Figure 3:**
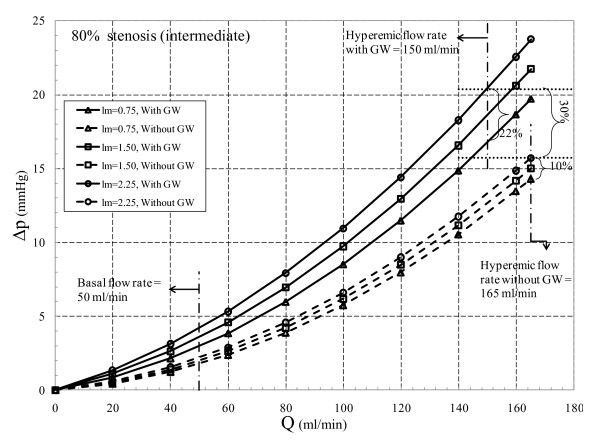
**80% area blockage Δp vs. Q characteristic**.

**Figure 4:**
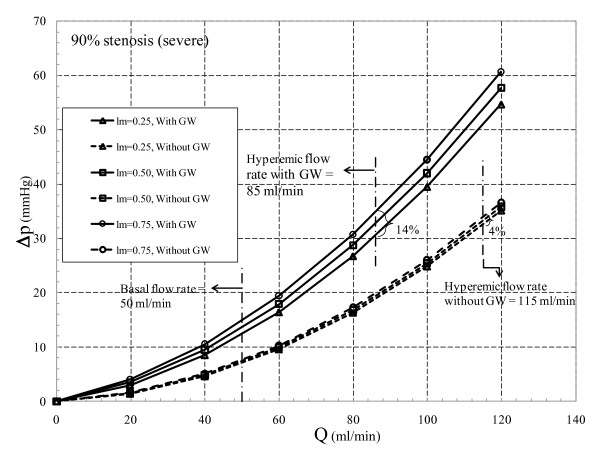
**90% area blockage Δp vs. Q characteristic**.

Total pressure drop across the stenosis is composed of loss due to momentum change and viscous loss. Viscous loss is linearly correlated to flow rate [equation (10)], while loss due to momentum change varies with the second power of flow rate [equation (11)]. The translesional pressure drop and its component variation with respect to flow rate for the three different area blockages are presented in Figures 2, 3, and 4. In addition, effects of throat length and GW insertion are also presented in these figures. It can be observed that increase in stenosis severity, GW insertion, and increase in lesion length increase the pressure drop along the plaque.

At basal flow and before insertion of GW for *64% area stenosis (moderate) *as shown in Figure 2 pressure drop increases from 0.59 to 0.86 mmHg, a 46% (= [{ (0.86-0.59)}/0.59] × 100) increase as *l_m _*increases from 1.5 to 4.5 mm. In hyperemic condition (180 ml/min), Δ*p *increases from 4.42 to 5.39 mmHg (22% increment) for the same *l_m _*range. In the presence of GW and for same *l_m _*range, pressure drop increases from 1.03 to 1.66 mmHg (61% increase) at basal flow and from 5.92 to 8.08 mmHg (36% increase) at hyperemic flow (173 ml/min). Moreover, at maximum throat length (*l_m_*= 4.5 mm) hyperemic Δ*p *increases by 48% (= [{(8.08-5.45)}/5.45] × 100) due to GW insertion only. This confirms the obstruction effect of GW which has also been shown by Roy et al. [28].

The Δp-*Q *characteristic for *80% area stenosis (intermediate) *where *l_m _*ranges from 0.75 to 2.25 mm is shown in Figure 3. Without GW at basal flow (50 ml/min), Δ*p *increases from 1.76 to 2.20 mmHg (25% increase) as *l_m _*increases from 0.75 to 2.25 mm, while for hyperemic flow (165 ml/min) and same range of *l_m_*, Δ*p *increases from 14.30 to 15.72 mmHg (10% rise). As the GW is inserted at basal flow and hyperemic flow, for the same range of throat lengths, Δ*p *increases from 2.95 to 4.17 mmHg (41% rise) and 16.73 to 20.39 mmHg (22% rise), respectively. In the intermediate stenosis, the difference between Δ*p *for same flow rate but different *l*_m _is diminished in both without and with GW insertion when compared to moderate stenosis. Although, there is a relative increase in overall Δ*p *because of GW insertion, its dependency on *l*_m _is reduced as compared to 64% AS. As the throat length increases for 64% AS at hyperemic flow rates with and without GW, Δ*p *increases from 22% to 36%, respectively, while these percentages reduce to 10% and 22% for intermediate stenosis. Moreover, percentage pressure drop increase at hyperemic condition due to GW insertion (30%) is less than that for 64% AS (48%). This indicates a decreasing trend in sensitivity of Δ*p *to GW insertion in stenoses with higher severity.

As mentioned before from *diagnostic viewpoint*, intermediate stenosis is a clinically challenging case. GW diagnostic is widely used to assess lesion severity by measuring FFR under hyperemic condition. Insertion of GW adds extra resistance to flow which results in sharp rise in Δ*p *and consequently a reduction in FFR value. At the maximum *l*_m _of 2.25 mm, Δ*p *increases by 30% (= [{20.39-15.72}/15.72] × 100) as the result of GW insertion, which underestimates FFR value. In 80% AS with proximal pressure (*p*_a_) of 86 mmHg [28], distal pressure (*p*_d_) reduces from 70.5 to 66 mmHg as the GW is inserted. This would change FFR from 0.85 (= 70.5/86) to 0.77 (= 66/86). Thus, in the presence of GW, FFR shows values around the limiting condition of 0.75 which may lead to misdiagnosis of lesion severity. With this regard, the Δ*p*-*Q *curves can be helpful in interpreting Δ*p *values and consequently the FFR results.

Similar to Figures 2 and 3, Δ*p-Q *curve is also obtained for the *severe stenosis (90%) *case with throat lengths ranging from 0.25 to 0.75 mm (Figure 4). In the absence of GW with increase in lesion length, Δ*p *shows 11% increase (from 6.90 to 7.48 mmHg) at basal flow (50 ml/min) and 4% rise (from 32.48 to 33.81 mmHg) at hyperemic condition (115 ml/min). In the presence of GW and for the same range of throat lengths pressure drop increases by 20% (from 12.21 to 14.70 mmHg) at basal flow (50 ml/min) and 14% (29.75 to 33.99 mmHg) at hyperemic flow (85 ml/min). Moreover, for the maximum *l*_m _of 0.75 mm, hyperemic Δ*p *increases only by 0.5% (= [{33.99-33.81}/33.81] × 100) due to GW insertion. Hence Δ*p *variation in severely stenosed arteries has a weak dependency on GW insertion when compared to that of moderate and intermediate stenoses.

**Figure 5:**
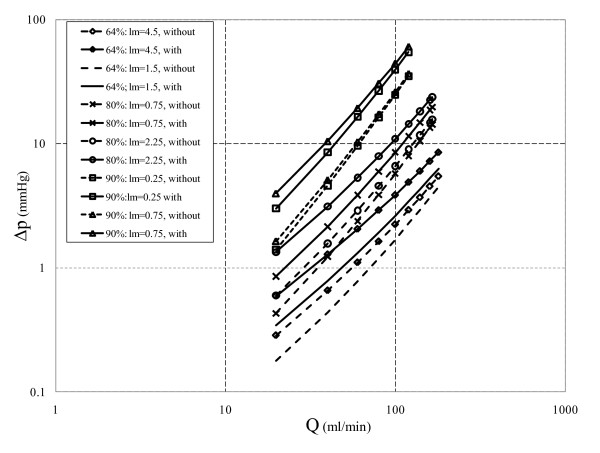
**All area blockages on log-log scale: Δp vs. Q characteristic**.

**Figure 6:**
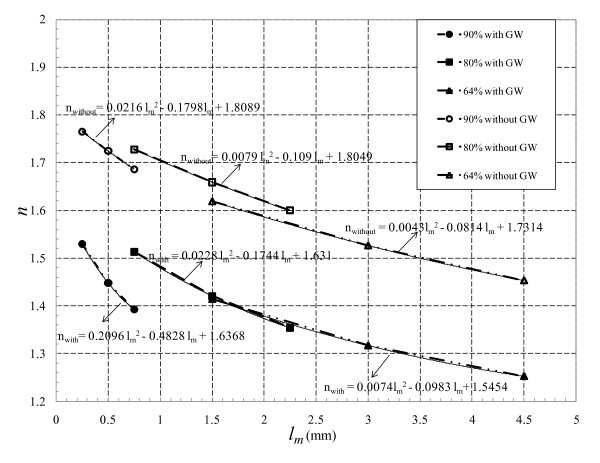
**Variation of slope *n *for all *l_m _*and percentage area blockages**.

Pressure drop is directly related to *l_m _*in both without and with GW cases, however, GW insertion increases Δ*p *non-linearly for all throat lengths. This can be elucidated by expressing the pressure drop-flow rate data in the *general form of *Δ*p = kQ^n^*. Here, *n *and *k *values can be obtained from the natural logarithm of the Δ*p-Q *curves. Log-log scale of Δ*p-Q *characteristic for the three AS at their corresponding throat lengths are shown in Figure 5. The resulting straight lines are regressed to find their corresponding slopes (*n*) and intercepts (*k*) that are summarized in Table 2. The values of *n *varies from 1 to 2, where *n *= 1 signifies purely viscous loss and *n*= 2 signifies purely loss due to momentum change. Hence, the value of *n *indicates the relative estimate of loss due to momentum change and viscous loss. Moreover, *n *shows the dependency of pressure drop to flow rate and varies with throat length, stenosis severity, and GW insertion. Figure 6 shows the variation in *n *values with respect to these parameters. Second order polynomials are fitted to the slope (*n*) *vs*. throat length (*l_m_*) data and the corresponding equations are given in the figure. As the lesion length increases *n *decreases due to the enhanced viscous effects (e.g., as *l_m _*increases from 1.5 to 4.5 mm in 64% AS with GW, the value of *n *decreases from 1.42 to 1.25). Moreover, at a fixed throat length, GW insertion increases the viscous forces and thus reduces the *n *values (e.g., at *l_m _*= 4.5 mm and 64% AS, with the insertion of GW *n *reduces from 1.45 to 1.25). However, increase in stenosis severity increases the fluid momentum and thus raises the *n *values (e.g., in the presence of GW, as the stenosis severity increases from 64% to 90%, *n *increases from 1.41 to 1.53).

**Table 2:** Slope and constant as per Δ*p *= *k × Q^n^*

Area stenosis		Without GW	With GW	Without GW	With GW	Without GW	With GW
	*l_m_*	**1.5**		**3.00**		**4.50**	
	
64%	*n*	1.6191	1.4145	1.5263	1.3168	1.4530	1.2523
	
	*k*	0.0001	0.0039	0.0017	0.0076	0.0028	0.0123

	*l_m_*	**0.75**		**1.5**		**2.25**	
	
80%	*n*	1.7276	1.5130	1.6592	1.4206	1.5997	1.3538
	
	*k*	0.0020	0.0081	0.0030	0.0142	0.0042	0.0217

	*l_m_*	**0.25**		**0.50**		**0.75**	
	
90%	*n*	1.7653	1.5292	1.7244	1.4478	1.6862	1.3926
	
	*k*	0.0070	0.0314	0.0086	0.0477	0.0103	0.0647

### 3.2 Loss Due to Momentum change and Viscous Loss

**Figure 7:**
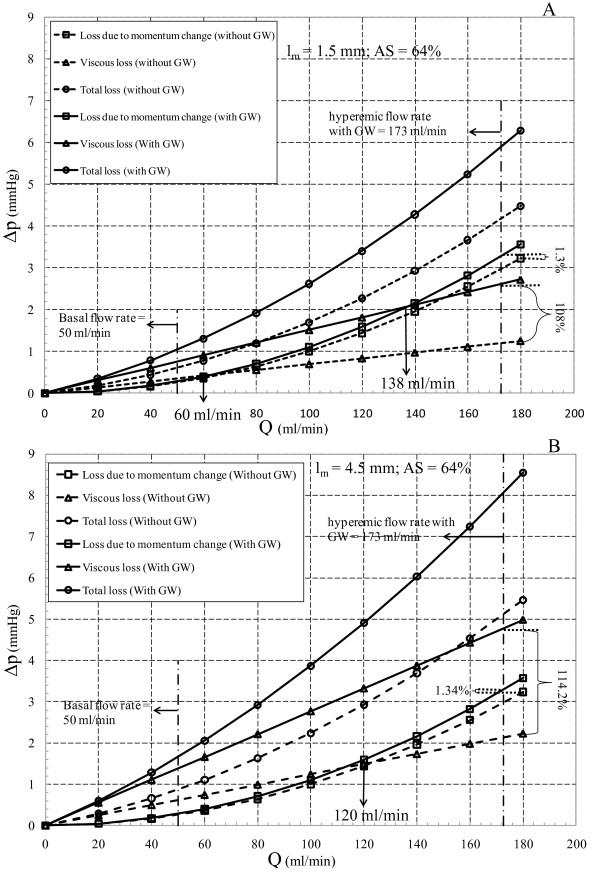
**Total drop along with loss due to momentum change and viscous loss for 64% area stenosis with (A) *l_m _*= 1.5 mm and (B) *l_m _*= 4.5 mm**.

**Figure 8:**
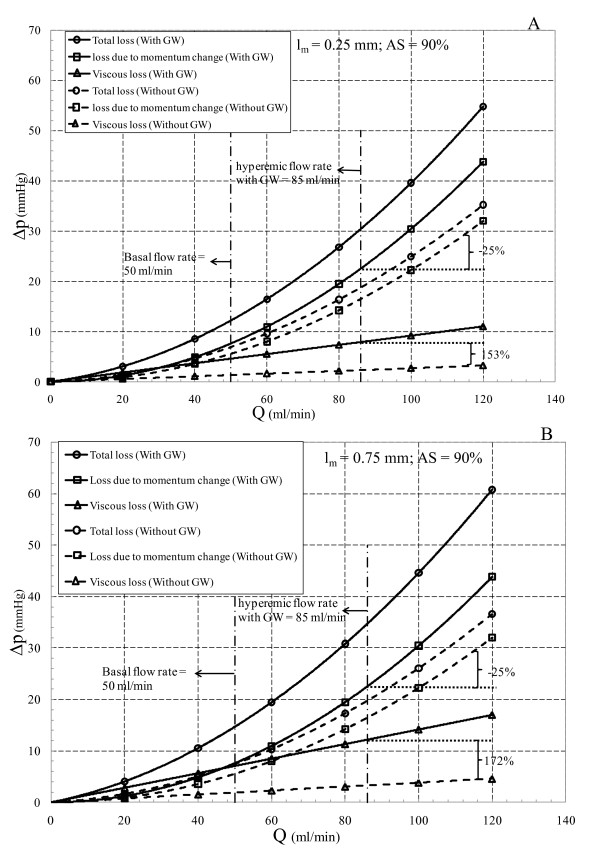
**Total pressure drop along with loss due to momentum change and viscous loss for 90% area stenosis with (A) *l_m _*= 0.25 mm and (B) *l_m _*= 0.75 mm**.

*Variation of loss due to momentum change and viscous loss *with flow rate for moderate and severe stenoses at their corresponding minimum and maximum lesion lengths are shown in Figures 7 and 8, respectively. Effects of GW insertion on the loss due to momentum change and viscous loss are also shown in these figures. Minimum and maximum throat lengths are, respectively, 1.5 and 4.5 mm for moderate stenosis, and 0.25 and 0.75 mm for the severe stenosis. As shown in Figure 7A, for moderate stenosis with minimum throat length (*l_m _*= 1.5 mm) before insertion of GW, loss due to momentum change is dominant and exceed the viscous loss at flow rate of 60 ml/min. Increasing the throat length to 4.5 mm (Figure 7B) reduces the dominancy range of loss due to momentum change, as they exceed the viscous loss at flow rate of 120 ml/min. However, at *l_m _*= 1.5 mm, insertion of GW elevates the viscous loss significantly (by 2.1 times), and viscous loss becomes dominant up to flow rate of 138 ml/min for the minimum throat length (see Figure 7A). For the maximum throat length (*l_m _*= 4.5 mm) as the GW is inserted, viscous effects, as can be seen in Figure 7B, remain dominant in the entire range of flow rates (viscous loss increases by 2.14 times in comparison with 1.34% rise in loss due to momentum change). This shows that viscous loss is dominant in moderate stenoses, which is consistent with the results of Young et al. [29].

In contrast to moderate stenoses, loss due to momentum change is dominant in the entire range of physiological flow rates (greater than 50 ml/min) for severe stenoses regardless of GW insertion, as shown in Figure 8. It is interesting to note that for the 90% AS, by inserting GW, loss due to momentum change for both minimum (*l_m _*= 0.25 mm) and maximum (*l_m _*= 0.75 mm) throat lengths decrease by 25% with similar pressure drop values (from 29.4 to 22 mmHg). However, viscous loss increases by 2.53 times for *l_m _*= 0.25 mm (from 3 to 7.8 mmHg) and by 2.72 times for *l_m _*= 0.75 mm (from 4.4 to 12 mmHg). It is noteworthy that regardless of this reduction in loss due to momentum change, it is still relatively more significant as compared to the viscous loss. Thus, severe stenoses can be categorized as momentum dominated plaques, which is also consistent with the results of Young et al. [29].

It should be noted that inserting GW in all cases increases the viscous loss relatively more than the loss due to momentum change, however, based on the stenosis severity, one of these losses becomes dominant. For example in the moderate stenoses the viscous loss tends to dominate the loss due to momentum change, while this trend is reversed in the severe stenoses. Therefore, insertion of GW decreases *n *in Δ*p *= *kQ^n ^*relationship (see Table 2). Furthermore, it is noteworthy that regardless of stenosis severity, changes in lesion length only affect the viscous loss.

**Figure 9:**
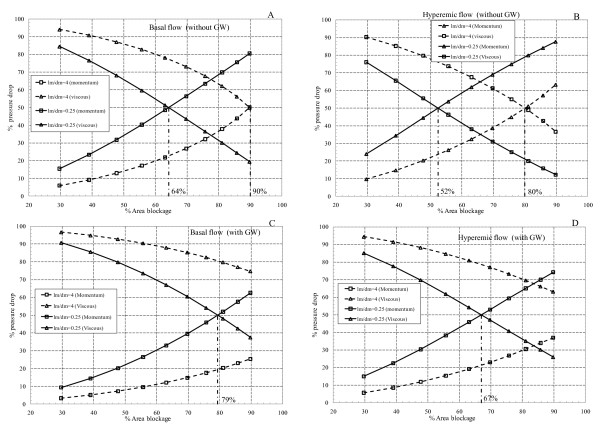
**Percentage momentum and viscous pressure drops for various *l_m_/d_m _*ratios for (A) basal flow (without GW); (B) hyperemic flow (without GW); (C) basal flow (with GW); (D) hyperemic flow (with GW)**.

Effects of flow rate and lesion length on the dominancy of loss due to momentum change and viscous loss are already discussed. Combined effects of stenosis severity (ranging from 30% to 90% AS), throat length to diameter ratio (*l_m_/d*_m_= 0.25 and 4.0), and GW insertion on the percentage viscous and momentum pressure drops are analyzed in Figures 9A, 9B, 9C, and 9D. Percentage pressure drop due to viscous loss (or loss due to momentum change) is defined as the ratio of viscous loss (or loss due to momentum change) to the total pressure drop. These figures are plotted at three constant flow rates corresponding to basal flow (50 ml/min), hyperemic flow in severe stenosis with GW (85 ml/min), and hyperemic flow in severe stenosis without GW (115 ml/min).

The *transitional percent area blockage *is defined as the point where loss due to momentum change overcomes viscous loss. For basal flow before insertion of GW and for *l_m_/d*_m _= 0.25 and 4.0 as shown in Figure 9A, transitional percent area blockages are 64% and 90%, respectively. As the flow rate increases to hyperemic condition these values reduce to 52% and 80%, respectively (Figure 9B). This reduction is due to higher dependency of loss due to momentum change to the second power of flow rate, while viscous loss is linearly related to flow rate. Thus transitional percent area blockages are reduced as flow is increased. Moreover, for *l_m_/d*_m _= 0.25 and before insertion of GW loss due to momentum change is dominant for moderate to severe stenoses, while for the *l_m_/d*_m _= 4.0 viscous loss is dominant even for severe stenoses. Furthermore, by insertion of GW (Figures 9C and 9D) viscous loss is dominant at both basal and hyperemic flow rates in the entire range of area stenoses for *l_m_/d*_m _= 4.0 However, for *l_m_/d*_m _= 0.25 the transitional percent area blockage are 79% and 67% at basal and hyperemic flow rates, respectively. These values are higher than the corresponding transitional area blockages for *l_m_/d*_m _= 0.25 without GW. This again shows that GW insertion contributes more to the rise in viscous loss rather than loss due to momentum change.

## 4. Discussion

The purpose of this study is to acquire a more fundamental understanding regarding the effect of GW insertion, lesion length, and stenosis severity on the total pressure drop across a stenosed coronary artery. Mean pressure drop along the stenosis is obtained under steady flow and Newtonian fluid assumptions. Contribution of the loss due to momentum change and viscous loss to the overall pressure drop along the lesion is also investigated.

**Figure 10:**
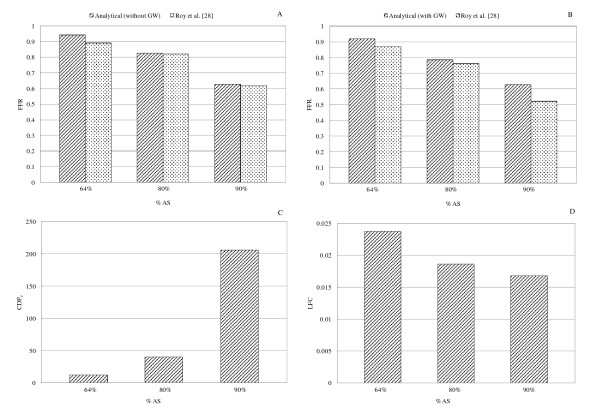
**Comparison of analytically obtained FFR values with the corresponding numerical results **[28]**without GW (A) and with GW (B) under hyperemic condition**. Variation of analytically obtained CDP_e _(C) and LFC (D) in the presence of GW for different stenosis severities. Here, for all the cases, the GW diameter is 0.35 mm.

Having obtained the translesional pressure drop (Δ*p *= *p_a_-p_d_*) from this analytical approach, and adopting proper pressure proximal to stenosis (*p_a_*),  can be calculated. Here, *p_a _*values for 90%, 80%, and 64% AS are taken as 89, 86, and 84 mmHg [28], respectively. Recently, our group has proposed two new diagnostic parameters namely pressure drop coefficient (CDP_e_) and lesion flow coefficient (LFC). These new diagnostic parameters are based on the fluid dynamic principles and can better diagnose the stenosis severity [30].  is defined as the ratio of mean translesional pressure drop to the proximal dynamic pressure , while  is defined as the ratio of percent area stenosis (1-κ) to the square root of the throat pressure drop coefficient . The analytical method can provide enough information to evaluate these diagnostic parameters (FFR, CDP_e_, and LFC) as shown in Figure 10. The analytically obtained FFR values are compared with the available numerical results [28] in the absence (Figure 10A) and presence (Figure 10B) of GW for different stenosis severity. It is noteworthy that FFR obtained analytically shows a trend similar to that of CFD results. Moreover, the percentage difference between FFR values obtained from the analytical and numerical approaches is around 5% (e.g. for 64% AS with GW: 4.6% [= (0.91-0.87)/0.87] × 100) for all the cases, except for the 90% area stenosis with GW which shows 21% ( =[(0.63-0.52)/0.52] × 100) difference. Also, variations of CDP_e _and LFC for different stenosis severities in the presence of GW are shown in Figures 10C and 10D, respectively. CDP_e _and LFC are not compared with any numerical or experimental results since these parameters have not been previously reported for the same geometries and flow rates considered in this study. It is observed that the increase in stenosis severity results in an increase in CDP_e _and reduction in the LFC which, in general, is consistent with the previous *in vivo *studies [30]. However, more studies need to be performed on the ability of this analytical approach in evaluating these new diagnostic parameters. In addition to the diagnostic parameters, obstructive effect of GW also can be measured using this analytical approach. Obstructive effect of GW can be quantified by the absolute difference of the pressure drop values between with and without guidewire cases. This effect results in 2 (= |7-5|), 3.5, and 1.2 mmHg changes in pressure drop at hyperemic condition for 64%, 80%, and 90% area stenosis, respectively.

**Figure 11:**
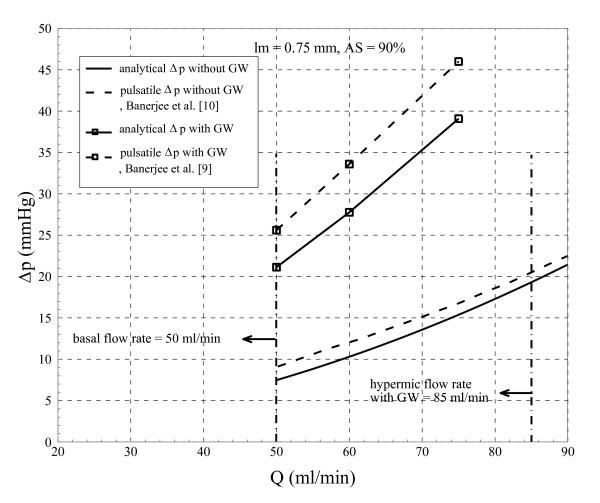
**Comparison of the current results for 90% area stenosis with pulsatile results from Banerjee et al**. [9,10].

Steady flow assumption is one of the major limitations of this analytical approach which neglects time dependent phenomena such as transient shear layer growth in the throat section and time varying recirculation zone distal to plaque. However, previous studies [9,10] have shown that mean translesional pressure drop for severe stenosis can be obtained within 20% error of pulsatile calculations. It should be mentioned that due to the small magnitude of pressure drop for the moderate stenosis (i.e. for mean flow rate of 180 ml/min, the analytical and numerical translesional pressure drops are 3.7 and 5.5 mmHg, respectively) the percentage difference between numerical and analytical results is relatively higher (33% = [(5.5-3.7)/5.5] × 100) as compared to the intermediate and severe stenoses, which will be discussed later. However, this higher percentage difference will not affect the diagnostic assessments (FFR values for the analytical and numerical approaches are 0.96 {= 1-3.7/84} and 0.93, respectively). Therefore, for intermediate to severe stenoses steady assumption can provide valuable information on hemodynamic parameters such as pressure drop under clinical setting. To address this point, as shown in Figure 11 the analytically obtained translesional pressure drop is compared to pulsatile result from Banerjee et al. [9,10]. They have used the same geometry and dimensions that are considered here for 90% AS with throat length of 0.75 mm, however, the GW diameter is 0.46 mm and not 0.35 mm. It can be seen that the analytical approach at most results in 14% to 17% error in the presence of GW (GW diameter of 0.46 mm), while for without GW the error ranges from 2% to 18%.

It should be mentioned that the translesional pressure drop obtained through the analytical approach is lower than the ones from the numerical calculations. This variation in the pressure drop is due to the simplified assumptions such as steady state and Newtonian fluid. However, this variation can be reduced by using a correction factor that can account for such limitations. In our previous studies on the translesional pressure drop for moderate and severe stenoses in the presence of GW [2,9], the ratio of the pressure drop for pulsatile and steady state flows under hyperemic condition is determined to be 1.16. The analytical pressure drop can be multiplied by this factor to obtain the corrected pressure drop. Table 3 shows the analytically and numerically obtained pressure drops [28] along with the corrected pressure drops and the corresponding FFR values under hyperemic condition. It can be seen that the percentage difference between the numerical and analytical pressure drops has significantly reduced by using this correction factor. For example for 64% area stenosis this difference has reduced from 29.3% (= [(9.9-7)/9.9] × 100) to 17.9% (= [(9.9-8.3)/9.9] × 100), while similar reduction has been observed for 80% (11.4% to 2.8%) and 90% AS (20.9% to 8.2%). Also as shown in the Table 3, the corrected pressure drops have reduced the difference between the corresponding corrected analytical and the numerical FFR values. For 64% AS this difference has reduced from 4.5% (= |(0.88-0.92)/0.88| × 100) to 2.3% (= |(0.88-0.9)/0.88| × 100), while a similar trend is also observed for 80% (from 3.8% to 1.3%) and 90% AS (19.2% to 7.7%).

**Table 3:** Numerical [28], analytical, and corrected analytical pressure drops along with the corresponding FFR values for different stenosis severity at hyperemic flow rate (Q)

AS	*l_m _*(mm)	Q (ml/min)	*p_a _*(mmHg)	Δp_numerical_	Δp_analytical_	Δp_corrected _= 1.16 × Δp_analytical_	FFR_numerical_	FFR_analytical_	FFR_corrected_
64%	3	173	84	9.9	7	8.13	0.88	0.92	0.9

80%	0.75	150	86	18.9	16.74	19.42	0.78	0.81	0.77

90%	0.75	85	89	43	34.02	39.46	0.52	0.62	0.56

The GW diameter is 0.35 mm.

In addition to steady state assumption, considering blood as a Newtonian fluid is another limitation of this study. Blood viscosity may affect the total pressure drop in the viscous dominated regions. From the results of this work it can be concluded that GW insertion tends to increase the viscous loss more than the loss due to momentum change (see Figures 7 and 8). However, due to high shear rate in the converging and throat sections, non-Newtonian behavior of blood is of minor importance. The adverse pressure gradient results in recirculation zone distal to the plaque where the non-Newtonian behavior of blood becomes important. However, previous studies have shown that Newtonian assumption has lesser influence on flow field in medium to large sized arteries such as coronary artery [31].

In previous studies [10,32,33] the occurrence of shear layer instabilities has been observed for intermediate to severe stenoses. Shear layer instability is considered as low Reynolds number turbulence flow phenomenon which can be observed experimentally, but are not easy to detect by numerical computations (may need a refined mesh and higher order numerical schemes). It should be mentioned that in this study the throat Reynolds number (*Re_m_*) is limited to 734 (where  and, ) for the limiting case (i.e. 90% area stenosis without GW at hyperemic flow condition). Therefore, the authors are assuming the flow regime to be laminar. However, although laminar assumption is valid for the cases considered in this study, there are still chances of occurrence of shear layer instabilities in physiological flows and experimental studies due to possible disturbances in the cardiac pulse and irregularities in plaque anatomy. It should be noted that the current analytical approach is based on laminar flow assumption, and thus, cannot account for the shear layer instabilities which is one of the limitations of this work.

## 5. Conclusion

Translesional pressure drops in stenosed coronary artery with different area blockages with and without GW presence are studied. Pressure drop-flow rate characteristics are obtained analytically for different area blockages (64%, 80%, and 90%) with different throat lengths. Variations in lesion length primarily affect the viscous loss. However, this effect diminishes as the stenosis severity increases from moderate to intermediate stenoses. In the severe stenoses, effect of lesion length is almost negligible.

Similar to lesion length effect, insertion of GW increases the viscous loss significantly. In moderate stenoses, viscous effects in the presence of GW can surpass the loss due to momentum change in the entire range of flow rates. In contrast, for the severe stenoses although the GW increases the viscous effects, the loss due to momentum change is completely dominant in the entire range of flow rates. It is noteworthy that insertion of GW, as compared to without GW case, increases the hyperemic pressure drop in the moderate to intermediate stenoses significantly. However, for the severe stenosis GW insertion has a negligible effect on the hyperemic translesional pressure drop. This finding which is in agreement with the previous study of Verberne et al. [34] might be due to appreciable reduction in flow rate as compared to without GW case. Moreover, insertion of GW increases the dominancy of viscous losses regardless of ste-nosis severity which can be observed with the reducing values of *n *(comparing without and with GW cases) in Table 2.

Also, total translesional pressure drop can be written in the form of Δ*p *= *kQ^n^*, in which *n *varies between 1 and 2, which are, respectively, the limits for viscous dominated and momentum dominated losses. Therefore, *n *can be used to assess the contribution of these two types of losses to the total pressure drop, and accordingly lesions can be cate-gorized into different groups of stenoses. Translesional pressure drop in the newly proposed diagnostic parameters is scaled either by viscous losses [4] or losses due to momentum changes [3]. Thus, evaluating *n *for specific flow rate and stenosis geometry can provide information on appropriate and accurate scaling approach for the diagnostic parameters. For the moderate stenosis with *n *values closer to 1, pressure drop may be scaled by viscous losses (or linear function of flow rate). However, for intermediate to severe stenosis with *n *values closer to 2, losses due to momentum changes (quadratic function of flow rate) can provide a better scaling for translesional pressure drop.

Moreover, pressure drop values obtained using this approach are comparable to the corresponding CFD results published in literature. Results of this approach can be further improved by modifying the current formulation to include a correction factor that can account for the pulsatile nature of coronary flows as well as the non-Newtonian behavior of blood. Also, with further improvements in clinical techniques such as QCA and Doppler flow catheters, this method has a potential to provide a quick evaluation of pressure drop and FFR values under bedside condition in the cardiac catheterization lab.

## Nomenclature

*A *= flow cross-sectional area (m^2^),, CFR = coronary flow reserve, *d_e _*= proximal vessel diameter (m), *d_i _*= catheter (or guidewire) diameter (m), *d_m _*= throat diameter (m), *d_o _*= mean vessel diameter (m), *f*= mean wall shear force = *2πrlτ_w _*(N), *F *= flow resistance, FFR = fractional flow reserve, I_s _= shear force integral, *l_c _*= length of converging region (m), *l_m _*= length of throat region (m), *l_r _*= length of diverging region (m), *p *= mean pressure (mmHg), *p_a _*= pressure proximal to the stenosis (mmHg), *p_d _*= pressure distal to the stenosis (mmHg), *p_r _*= Recovered pressure distal to stenosis (mmHg), Δ*p *= overall mean pressure drop, Δ*p_viscous _*= Overall pressure drop due to viscous loss (mmHg), Δ*p_momentum _*= Overall pressure drop corresponding to loss due to momentum change (mmHg), Δ*p_cm _*= pressure drop across constriction region (mmHg), Δ*p_m _*= viscous pressure drop in throat region (mmHg), Δ*p_rm _*= pressure recovery in divergent and distal region (mmHg), *Q*= volume flow rate (ml/min), *r *= radial distance (m), *r_o _*= mean vessel radius (m), *t *= time (s), *ū *= average axial velocity (m/s), *x *= axial distance (m), *β *= momentum coefficient, *λ *= conical half angle of constriction, *μ *= blood dynamic viscosity (Kg/m-s), *υ *= blood kinematic viscosity (μ/ρ) (cP), *ρ *= blood density (Kg/m^3^), *P_o_*= vessel perimeter (m), *τ_w _*= wall shear stress (N/m^2^), *T *= period of cardiac cycle (s), , , 

Subscripts:

*e*= proximal vessel, *i *= catheter, *m *= throat region, *o *= vessel, *r *= recovery point, *w *= wall condition

Superscripts:

(~) = time average over cardiac cycle, (-) = mean flow

## Competing interests

The authors declare that they have no competing interests.

## Authors' contributions

ERJ and KK implemented the mathematical analyses, numerical calculations with equal contribution in this work. And participated in the sequence alignment and drafted initial versions of the manuscript under supervision of RKB. LHB provided technical insights into this research during numerical data analysis and critically revised the draft. All authors read and approved the final manuscript.
